# Choroidoscleral Interface Irregularity Index: A novel optical coherence tomography-based parameter in patients with epiretinal membrane

**DOI:** 10.1038/s41598-020-57656-w

**Published:** 2020-01-20

**Authors:** Mirinae Kim, Yohan Lee, Rae-Young Kim, Jae Hyuck Kwak, Young-Hoon Park

**Affiliations:** 1 Department of Ophthalmology and Visual Science, College of Medicine, The Catholic University of Korea, Seoul, South Korea; 2 School of Electrical and Computer Engineering, Seoul National University, Seoul, South Korea; 3 Catholic Institute for Visual Science, College of Medicine, The Catholic University of Korea, Seoul, South Korea

**Keywords:** Prognostic markers, Retinal diseases

## Abstract

This study aimed to assess the regularity of the choroidoscleral interface (CSI) using a novel parameter, CSI irregularity index, before and after epiretinal membrane (ERM) surgery. This study included 36 patients with idiopathic ERM who underwent pars plana vitrectomy and ERM removal. All subjects underwent ocular examinations at baseline and at 1, 2, 4, and 6 months after surgery. The regular contour of the CSI was found in 14 patients (38.9%); mean CSI irregularity index was 14.84 ± 11.01 in this group. The irregular contour of the CSI was found in 22 patients (61.1%); mean CSI irregularity index was 33.96 ± 20.64 in this group. The CSI irregularity index decreased gradually after ERM surgery, and was correlated with postoperative best-corrected visual acuity. The CSI irregularity index could serve as a surrogate marker to quantitatively represent the CSI morphology. We observed the gradual decrease of the CSI irregularity index after ERM surgery in quantitative manner. This study showed correlations between the CSI irregularity index and visual outcomes after ERM surgery. Our results suggest that the CSI irregularity index might be an intuitive anatomic indicator of the CSI and might be useful as a possible prognostic marker for patients undergoing ERM surgery.

## Introduction

Epiretinal membrane (ERM) is one of the most common retinal diseases, characterized by fibrocellular tissue proliferation on the inner retinal surface^1–3^. Surgical removal of ERM is the only option in eyes that require treatment^4^. Surgical removal of ERM in symptomatic patients can improve visual acuity (VA) by more than 2 Snellen lines and reduce metamorphopsia in 70-90% of cases^5–8^. However, in some cases, VA improvement may be limited after successful ERM removal^9^. Thus, previous studies have attempted to identify prognostic factors that indicate which patients exhibit a better outcome following ERM surgery^9–16^. Many studies have focused on the relationship between central foveal thickness and visual outcome after ERM surgery; however, the results have been inconsistent^12–14^. Outer retinal layer damage, including disruption of the ellipsoid zone (EZ) or cone outer segment tip (COST), has been associated with poor visual outcomes after ERM surgery^13,15^. In recent years, inner retinal deformation has been suggested as an important prognostic factor in ERM. Inner retinal irregularity index and inner retinal layer thickness were reportedly associated with poor visual outcomes; in contrast, retinal contraction was associated with good visual outcomes after ERM surgery^10,11,16^. However, a recent systematic review revealed that many discrepancies exist between studies; thus, an overall prognosis prediction model is still needed^17^.

The role of the choroid in the pathogenesis of ERM is not well-known. However, recent studies have suggested that ERM might involve more complex etiopathology than solely vitreoretinal interface changes^14,18,19^. In previous reports, the regularity of the choroidoscleral boundary was evaluated in macular hole^20^ and ERM^18,19^. Michalewski *et al*.^19^ suggested that there may be a relationship between choroidal thickness and structure and the presence of idiopathic ERM. With recent advances in optical coherence tomography (OCT), precise visualization of the choroid and the choroidoscleral interface (CSI) have become possible. In the literature, irregular CSI has frequently been reported in patients with ERM; moreover, patients with irregular CSI recovered VA more rapidly after ERM surgery^18,19^. However, there is limited information regarding CSI before and after ERM surgery; notably, previous researchers have evaluated CSI solely in a qualitative manner.

The aim of this study was to quantitatively assess the regularity of CSI using a novel parameter called CSI irregularity index. Additionally, this study evaluated the effectiveness of the CSI irregularity index as a predictive prognostic marker for postoperative outcome in patients with idiopathic ERM.

## Results

**Figure 1:**
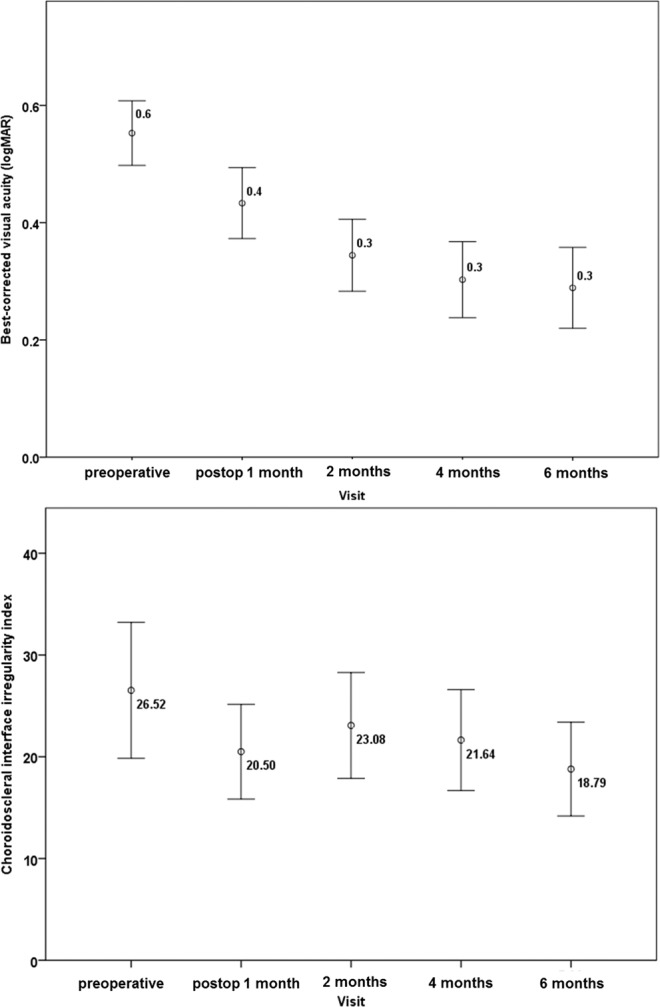
Graphs showing temporal changes in the best-corrected visual acuity and choroidoscleral interface irregularity index. The mean best-corrected visual acuity (BCVA) improved gradually (*P* < 0.001), and the choroidoscleral interface (CSI) irregularity index decreased gradually after epiretinal membrane (ERM) surgery (*P* < 0.001). Transient, sudden decline of the CSI irregularity index at 1 month after ERM surgery might be due to residual gas tamponade effect.

We analyzed 36 eyes of 36 patients. The demographic and clinical characteristics of the patients with ERM are depicted in Table 1. Ten patients (27.8%) were male and 26 (72.2%) were female. The bowl-shaped (regular or concave) contour of the CSI was found in 14 patients (38.9%); the mean CSI irregularity index was 14.84 ± 11.01 in this group. The inflective (irregular or S-shaped with ≥1 inflection point) contour of the CSI was found in 22 patients (61.1%); the mean CSI irregularity index was 33.96 ± 20.64 in this group. However, there were no significant differences in central foveal thickness, central choroidal thickness, or EZ/COST defect grade between the two groups, according to CSI morphology (*P* = 0.774, 0.094, and 0.130, respectively). Further, there were no significant differences in sex, age, presence of diabetes or hypertension, symptom duration, axial length, intraocular pressure, best-corrected visual acuity (BCVA), or refractive error between the two groups (all *P* > 0.05). Figure 1 shows temporal changes in BCVA and the CSI irregularity index. The mean BCVA improved gradually, the CSI irregularity index decreased gradually after ERM surgery (*P* < 0.001 for trend).

**Table 1:** Demographics and clinical characteristics of patients with epiretinal membrane.

	Total (N = 36)	Bowl-shaped (N = 14)	Inflective (N = 22)	*P* value
Age (year)	65.0 ± 7.0	65.8 ± 8.3	63.9 ± 6.1	0.451*
Sex (male)	10 (27.8%)	4 (28.6%)	6 (27.3%)	0.962†
Diabetes mellitus	8 (22.2%)	3 (21.4%)	5 (22.7%)	0.898†
Hypertension	11 (30.6%)	5 (35.7%)	6 (27.3%)	0.692†
Symptom duration (years)	2.45 ± 1.57	2.96 ± 2.03	2.11 ± 1.12	0.114*
Axial length (mm)	23.84 ± 1.27	24.08 ± 1.22	23.69 ± 1.30	0.381*
Intraocular pressure (mmHg)	14.00 ± 2.81	14.29 ± 2.92	13.86 ± 2.71	0.661*
Best-corrected visual acuity (logMAR)	0.55 ± 0.16	0.53 ± 0.14	0.57 ± 0.18	0.485*
Refractive error (spherical equivalent)	−0.40 ± 1.78	−0.62 ± 1.64	−0.26 ± 1.87	0.560*
Central foveal thickness (μm)	487.02 ± 61.80	483.0 ± 58.98	489.18 ± 64.73	0.774*
Central choroidal thickness (μm)	215.03 ± 75.40	188.93 ± 85.21	232.18 ± 65.04	0.094*
Inner retinal irregularity index	1.25 ± 0.09	1.22 ± 0.06	1.26 ± 0.11	0.250*
**Choroidoscleral interface morphology**				
Bowl-shaped (regular)	14 (38.9%)			
Inflective (irregular)	22 (61.1%)			
Choroidoscleral interface irregularity index	26.52 ± 19.74	14.84 ± 11.01	33.96 ± 20.64	0.001*
EZ/COST defect grade	1.00 ± 0.61	0.86 ± 0.36	1.14 ± 0.71	0.130*

EZ/COST, ellipsoid zone/cone outer segment tip; logMAR, logarithm of the minimum angle of resolution.

*P value determined by independent t-test.

^†^*P* value determined by Fischer’s exact test.

Table 2 demonstrates the correlation of the predictive prognostic markers with BCVA at each follow-up time point. Inner-retinal irregularity index showed correlations with BCVA at all-time points (*P* = 0.012, *P* = 0.049, *P* = 0.045, *P* = 0.017, *P* = 0.035 for before surgery and 1, 2, 4, 6 months postoperatively, respectively). EZ/COST defect grade was correlated with BCVA solely at baseline (*P* = 0.007). CSI irregularity index was correlated with BCVA at 4 and 6 months after ERM surgery (*P* = 0.042 and *P* = 0.013, respectively). However, central foveal thickness and central choroidal thickness did not show any correlation with BCVA at any time points (all *P* > 0.05).

**Table 2:** Correlations of possible predictive prognostic markers and visual acuity in patients with epiretinal membrane.

	BCVA				
	Before surgery	POD 1 m	POD 2 m	POD 4 m	POD 6 m
Central foveal thickness	*P* = 0.749 r = −0.055	*P* = 0.560 r = 0.100	*P* = 0.135 r = 0.254	*P* = 0.409 r = 0.142	*P* = 0.250 r = 0.197
Central choroidal thickness	*P* = 0.082 r = 0.294	*P* = 0.135 r = 0.254	*P* = 0.591 r = 0.093	*P* = 0.205 r = 0.217	*P* = 0.884 r = 0.025
Inner-retinal irregularity index	*P* = 0.012 r = −0.064	*P* = 0.049 r = 0.099	*P* = 0.045 r = 0.123	*P* = 0.017 r = 0.266	*P* = 0.035 r = 0.115
EZ/COST defect grade	*P* = 0.007 r = 0.441	*P* = 0.173 r = 0.018	*P* = 0.808 r = 0.042	*P* = 0.602 r = 0.090	*P* = 0.478 r = 0.122
Choroidoscleral interface irregularity index	*P* = 0.917 r = −0.018	*P* = 0.450 r = −0.130	*P* = 0.263 r = 0.191	*P* = 0.042 r = 0.341	*P* = 0.013 r = 0.409

BCVA, best-corrected visual acuity; EZ/COST, ellipsoid zone/cone outer segment tip; POD, postoperative days.

Table 3 shows the results of a regression analysis of the correlation of preoperative parameters with 6-month postoperative BCVA. Univariate regression analysis showed that inner-retinal irregularity index (r = 0.536, *P* = 0.001), EZ/COST defect grade (r = 0.331, *P* = 0.048), and CSI irregularity index (r = 0.272, *P* = 019) exhibited correlations with postoperative BCVA. Multiple regression analysis showed that inner-retinal irregularity index was strongly correlated with postoperative BCVA (r = 0.498, *P* = 0.003). Central choroidal thickness, EZ/COST defect grade and CSI irregularity index did not exhibit significance in the multiple regression analysis model (*P* = 0.335, *P* = 0.107 and *P* = 0.198, respectively). The factors are independent each other because there is low degree of intercorrelation (multicollinearity) in the multiple regression analysis.

**Table 3:** Linear regression analysis of factors associated with best-corrected visual acuity at 6 months postoperatively.

	Univariate		Multivariate	
	Standardized regression coefficient	*P* value	Standardized regression coefficient	*P* value
Preoperative best-corrected visual acuity	0.229	0.180		
Central foveal thickness	0.264	0.120		
Central choroidal thickness	0.106	0.539	−0.155	0.335
Inner-retinal irregularity index	0.536	0.001*	0.498	0.003*
EZ/COST defect grade	0.331	0.048*	0.266	0.107
Choroidoscleral interface irregularity index	0.272	0.019*	0.084	0.198

EZ/COST, ellipsoid zone/cone outer segment tip.

For each observer, there was a high intra-observer agreement for the CSI irregularity index (ICC = 0.926; 95% confidence interval = 0.901–0.945). Furthermore, there was a high inter-observer agreement between the 2 investigators for the CSI irregularity index (ICC = 0.862; 95% confidence interval = 0.820–0.896).

## Discussion

To the best of our knowledge, this study is the first to quantitatively analyze the choroidoscleral interface. We observed reduction of CSI irregularity after ERM surgery, and the CSI irregularity index was correlated with postoperative visual outcome.

Inner-retinal layer distortions other than outer retinal changes have gained attention for predicting the visual prognosis after ERM surgery, because ERM is primarily an inner retinal disease^10,11,16^. With the advent of the SS-OCT device, changes in choroidal structures have been assessed in recent studies^18,21,22^. ERM might lead to choroidal thickening and choroidal vasculature changes through a variety of possible mechanisms. Tangential traction caused by epiretinal membrane can affect the retinal pigment epithelium and choroid. Another possible explanation of the choroidal changes is that the mechanical stretching of the RPE may increase the expression of vascular endothelial growth factor, which might lead to choroidal hyperpermeability or choroidal vascular rearrangement. Further, low-grade chronic inflammation might be present in the choroidal layer, which might cause choroidal vascular changes.

In this study, we analyzed correlations of the CSI irregularity index and several OCT-derived parameters in ERM, as well as visual outcomes after ERM surgery. Our study showed that CSI irregularity index decreased gradually after ERM surgery. Transient, sudden decline of the CSI irregularity index at 1 month after ERM surgery might be due to residual gas tamponade effect. In previous reports, the regularity of the choroidoscleral boundary was evaluated in macular hole^20^ and ERM^18,19^. Michalewska *et al*. reported that an irregular choroidoscleral boundary was more frequently observed in diseased eyes, such as those with ERM; moreover, patients with an irregular outer choroidoscleral boundary recovered visual function more rapidly after ERM surgery^18^. To quantitatively assess these findings, we developed a novel parameter called CSI irregularity index. In the regression analysis, the CSI irregularity index showed positive correlations with postoperative BCVA. However, this new parameter is not more robust than the inner-retinal irregularity index for prediction of visual outcome. We can utilize this index as one of multiple prognostic markers in ERM surgery. Excellent intraobserver and interobserver repeatability was observed for CSI irregularity index, as indicated by the ICC values.

In the macular area, the choroid typically is thickest at the subfovea; choroidal thickness decreases gradually toward the temporal side and more steeply toward the optic nerve^23–25^, exhibiting a bowl-shaped contour^26,27^. The CSI may either be regular or irregular, even in healthy eyes, particularly when temporal inflection of CSI occurs with focal thinning of the choroid^26^. Dolz-Marco and colleagues hypothesized that this inferotemporal inflection could be related to the inferior oblique muscle causing inward compression of the choroid^28^. In diseased eyes, higher CSI irregularity index (or an irregular CSI) might be result from focal dilation of choroidal vessels. This might be due to tractional force caused by the ERM itself. Another factor is the status of vitreous oxygenation after vitrectomy. After removal of the vitreous, which is oxygen-consuming tissue, the retina and choroid are exposed to a more highly oxygenated environment. These may result in reduction of choroidal thickness and CSI irregularity index after vitrectomy^18^.

Our study had some limitations. First, due to the nature of the retrospective study design, cataract surgery was performed at the surgeon’s discretion; thus, some patients underwent cataract surgery, while others did not. However, previous studies reported that cataract surgery did not affect visual prognosis after ERM surgery^10,16^. Second, gas tamponade with 14% perfluoropropane (C_3_F_8_) was conducted for each patient at the end of the surgery. The gas tamponade procedure was performed at the surgeon’s discretion to avoid immediate postoperative hypotony. Third, the follow-up period after ERM surgery was relatively short. Changes in retinal and choroidal structure and morphology could last up to 12 months after ERM surgery^29^. Fourth, the shape of the CSI could differ on the basis of the location of the OCT sections, even within a single patient. Furthermore, when measuring the CSI irregularity index, manual determination of the CSI should be conducted. There were many cases of artifacts in CSI tracking when using the automated segmentation layer line. In our study, two independent observers repeated the CSI tracking to ensure accuracy, and inter-observer agreement between the 2 investigators was high. However, this part should be supplemented in order to adopt this approach in clinical practice.

In conclusion, we developed a novel surrogate marker, the CSI irregularity index, for quantitative representation of CSI morphology. We quantitatively observed gradual reduction of the CSI irregularity index after ERM surgery. This study revealed correlations between the CSI irregularity index and visual outcomes after ERM surgery. Based on our results, the CSI irregularity index might constitute an intuitive anatomic indicator of the CSI and might be useful as one of multiple prognostic markers of ERM surgery.

## Methods

### Subjects

This was a retrospective cohort study of consecutive patients with idiopathic ERM who underwent uncomplicated pars plana vitrectomy and ERM removal between December 2016 and March 2018 at Seoul St. Mary’s Hospital in Korea. Exclusion criteria for the study were as follows: (1) secondary ERM (uveitis, diabetic retinopathy, retinal detachment, or retinal vein occlusion), (2) high myopia (axial length greater than 26.0 mm, or refractive error greater than −6.0 diopters), (3) eyes with a history of any intraocular surgery, ocular trauma, or laser treatment, (4) the presence of other retinal diseases, including glaucoma, age-related macular degeneration, or coexisting macular hole, and (5) media opacity that significantly affect OCT image. Patients were also excluded if they exhibited ERM in both eyes.

This study adhered to the tenets of the Declaration of Helsinki. Institutional Review Board (IRB)/Ethics Committee approval was obtained from the Catholic University of Korea. It waived the requirement to obtain informed consent because of the retrospective nature of the study.

### Surgical technique

One experienced vitreoretinal surgeon (Y.H.P.) performed all of the operations. A standard 3-port pars plana vitrectomy was performed using a 25-gauge trocar and cannula (Alcon Laboratories, Fort Worth, TX; Dutch Opthalmic Research Center, Zuidland, The Netherlands). In patients with a visually significant cataract, cataract operation was performed before vitrectomy. For each patient, ERM and the internal limiting membrane (ILM) were peeled with end-gripping forceps. ILM peeling was performed after staining of the ILM with 0.05% indocyanine green dye; the area of ILM peeling was as large as a 2- or 3-disc diameter, with the fovea as the center. At the end of the surgery, gas tamponade with 14% perfluoropropane (C_3_F_8_) was conducted for each patient.

### Ophthalmic examination

Demographic information medical histories were recorded before surgery. Postoperatively, patients were examined at 1, 2, 4, and 6 months. All subjects underwent ocular examinations that included BCVA evaluation (logarithm of the minimum angle of resolution [logMAR] scale), slit-lamp biomicroscopy, funduscopy, pneumatic tonometry, axial length measurement with partial coherence interferometry (IOL Master, Carl Zeiss Meditec, Jena, Germany) and OCT. OCT imaging was performed using a swept-source (SS)-OCT device (DRI Triton, Topcon, Tokyo, Japan).

### Optical coherence tomography image analysis

We performed SS-OCT examination on all study subjects. The detailed OCT acquisition methods were described in a previously published article^30^. We obtained a six-line radial pattern scan (1,024 A-scans) centered on the fovea from each eye. We performed OCT image acquisition between 2 and 5 PM to exclude known diurnal variations of choroidal thickness or choroidal vasculature.

We obtained retinal and choroidal thicknesses using in-built OCT software. The software automatically created thickness maps in accordance with ETDRS sectors and we used the central retinal and choroidal thicknesses of the innermost 1-mm circular area for analysis. For each OCT scan, all automated segmented layer lines could be manually adjusted to avoid possible segmentation errors.

The inner-retinal irregularity index was calculated as the length of the inner plexiform layer divided by the retinal pigment epithelial layer length using ImageJ software (version 1.51; National Institutes of Health, Bethesda, MD, USA), as described previously^10,16^. Disruption of the EZ/COST line was detected when there was a loss of a hyperreflective line at the fovea^31^. We graded the disruption of the EZ/COST line in accordance with the global disruption scale, as described previously^32,33^. Grade 0 was assigned when the line was intact, Grade 1 was assigned when the focal disruption of the line was 200 microns or less, and Grade 2 was assigned when the disruption was greater than 200 microns.

### Choroidoscleral interface irregularity index measurement

**Figure 2:**
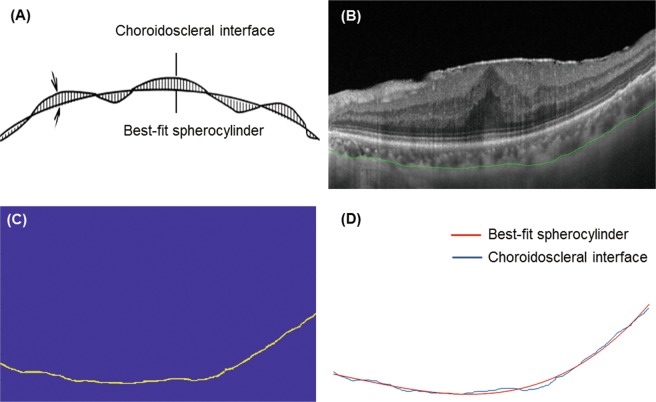
The definition and a representative image of measurement of the CSI irregularity index. (**A**) The hatched area between the choroidoscleral interface (CSI) on optical coherence tomography and its best-fit spherocylinder demonstrate the concept of the CSI irregularity index, which is defined as the weighted sum of differences at each measured point between the CSI on optical coherence tomographic image and its best-fit spherocylinder. (**B**) Before importing images into Matlab software, the CSI was manually delineated. (**C**) After removing background noise with the thresholding filter in Matlab software, the contour of the CSI was subsequently extracted. (**D**) The polynomial curve was used for curve fitting with the Curve Fitting Tool in Matlab software. The order of polynomial function was determined to identify the best approximation to the shape of the CSI. The Polyfit function was used to fit the coordinates via the least squares principle. CSI irregularity index = 12.6665.

We developed a new parameter to quantitatively assess the irregularity of CSI for SS-OCT images. CSI irregularity index is defined as the weighted sum of differences at each measured point between the CSI on optical coherence tomographic image and its best-fit spherocylinder. Briefly, the horizontal OCT scan image passing through the central fovea was imported into Matlab R 2017b software (MathWorks, Natick, MA, USA). Before importing images into Matlab software, the CSI was manually delineated by two masked observers (M.K. and R.Y.K.). At first, the graders judged the CSI irregularity in an intuitive manner. CSI may be either be bowl-shaped (regular) or follow the natural oval shape of the globe or be inflective (irregular)^18^. We compared the parameters according to these 2 groups. After importing images into Matlab software, the image was pre-processed to remove background noise, with the thresholding filter in Matlab used to differentiate intensity between background and the signal from the CSI. The contour of the CSI was subsequently extracted and curve-fitted using a third-order polynomial. The Polyfit function was used to fit the coordinates by following the least square principle. The polynomial curve was used for curve fitting with the Curve Fitting Tool in Matlab software. The order of polynomial function was determined to identify the best approximation to the shape of the CSI. In addition, the shape of the CSI was approximated as a smooth curve without any sharp point. The definition and a representative image of measurement of the CSI irregularity index are shown in Fig. 2.

### Reliability assessment

The CSI was independently assessed by two masked graders (M.K. and R.Y.K.). We evaluated both intra- and inter-observer reliability for assessing CSI irregularity index. The reliability of the CSI irregularity index was assessed by the absolute agreement model of the intraclass correlation coefficient (ICC). A high ICC value (ICC ≥ 0.8) indicates the good agreement between the investigators^34^.

### Statistical analysis

We expressed categorical data as absolute numbers, and continuous data as mean ± standard deviation (95% confidence interval). We conducted statistical analysis using the Statistical Package for the Social Sciences for Windows version 23.0 (SPSS Inc., Chicago, IL, USA). The normality of data distribution was confirmed using the Kolmogorov–Smirnov test. Demographic and clinical data of patients were compared by independent t-test, chi-square test, and Fisher’s exact test. To investigate the relationships between the BCVA and predictive prognostic factors, we used Spearman rank correlation coefficient for the central foveal thickness, central choroidal thickness, inner-retinal irregularity index, EZ/COST defect grade, and CSI irregularity index. Univariate and multivariate regression analysis were conducted to assess the correlation between the 6-month visual outcome and predictive prognostic factors. Statistical significance was assumed when *P* < 0.05.

## Data Availability

The datasets during and/or analyzed during the current study are available from the corresponding author on reasonable request.
